# MR imaging detection of adhesive capsulitis of the shoulder: impact of intravenous contrast administration and reader’s experience on diagnostic performance

**DOI:** 10.1007/s00256-022-03994-x

**Published:** 2022-02-28

**Authors:** Bernd Erber, Nina Hesse, Christian Glaser, Andrea Baur-Melnyk, Sophia Goller, Jens Ricke, Andreas Heuck

**Affiliations:** 1 Department of Radiology, University Hospital, LMU Munich, Marchioninistr. 15, 81377 Munich, Germany; 2 Radiologisches Zentrum München, Pippinger Str. 25, 81245 Munich, Germany

**Keywords:** MR imaging, Adhesive capsulitis, Intravenous contrast administration

## Abstract

**Objective:**

Correct identification of adhesive capsulitis of the shoulder (ACS) has an important impact on adequate therapy. The aim of our study was to investigate the influence of intravenous contrast administration and of reader’s experience on sensitivity and specificity of MRI in diagnosing ACS.

**Materials and methods:**

A total of 180 patients were included in a retrospective study: 60 subjects with at least 4 of 5 clinical signs of adhesive capsulitis of the shoulder and 120 patients with other shoulder diseases who underwent contrast-enhanced MRI. In a first session, only non-enhanced images and in a second session also contrast-enhanced (CE) series were independently evaluated by three radiologists with various levels of professional experience. Readers were blinded to all clinical information and had to rate the shoulder MRIs for absence or presence of adhesive capsulitis. Data analysis included McNemar’s test, *t* test, and *U* test (*p* < .05).

**Results:**

Using non-enhanced MRI, readers achieved a mean sensitivity of 63.9% and a mean specificity of 86.4%. By additional use of CE sequences, the mean sensitivity (85.5%) and the sensitivity for each reader increased significantly (*p* = .046, *p* < .01, *p* < .001, *p* = .045) while the improvement in mean specificity was not significant. Reader’s experience had a positive effect on sensitivity and specificity, which was in part but not consistently significant.

**Conclusion:**

The addition of CE sequences can significantly increase the sensitivity of MRI in the diagnosis of ACS. Reader’s experience has shown to be another important factor for the diagnostic outcome.

## Introduction

Adhesive capsulitis of the shoulder (ACS) is a common, but still poorly understood condition of the shoulder [1]. The terms “frozen shoulder” and “adhesive capsulitis” were first used by Codman (1934) and Neviaser (1945), respectively, to describe a painful limited range of motion (ROM) of the shoulder [2, 3]. ACS mostly occurs in the middle-aged population and women are more often affected than men [4, 5]. The disease is classified into an idiopathic primary form and a secondary form which is caused by previous trauma, surgery, or other diseases [6]. The course of ACS is divided into four stages and primary ACS is usually a self-limiting condition with a mean duration of symptoms between 18 and 24 months [3, 7, 8]. Among other diseases, the primary form may be associated with diabetes mellitus or autoimmune disorders [9]. Pathophysiologically, ACS is considered a fibrotic capsular disease with proliferation of fibroblasts and chronic inflammatory cells, predominantly in the rotator interval and the axillary recess [10].

Although there are no definite criteria, diagnosis of ACS is established primarily on the clinical findings of functional restriction of both active and passive shoulder motion [1]. An important role of imaging is to exclude other causes of painful limited ROM of the shoulder such as calcific periarthritis, osteoarthritis, or rotator cuff disease [1, 2, 11]. While the former two entities can be primarily ruled out using conventional radiographs, MRI is the method of choice for assessing soft tissue abnormalities [12]. Several studies sought for individual diagnostic signs of ACS in MRI; however, if not very conspicuous, they may be easily overlooked in routine imaging [13]. In particular, there are four anatomic regions to consider for MR imaging signs of ACS: the rotator interval, the axillary recess, the coracohumeral ligament, and the subcoracoid fat triangle [2, 11, 14–16]. A higher conspicuity associated with increased sensitivity and specificity in the detection of ACS was reported for additional intravenous (IV) administration of MR contrast medium [11, 16]. However, due to its more invasive nature, contrast-enhanced (CE) MRI has not been widely established yet in the diagnostic workup of ACS.

The aim of our study was to investigate the impact of CE sequences in addition to non-enhanced sequences on the MRI-based decision on the presence or absence of ACS in patients with shoulder symptoms and on the reader’s diagnostic confidence. In addition, we aimed to evaluate how reader’s experience may influence the diagnostic results.

## Methods

### Patients

Between January 2019 and December 2020, sixty patients with clinical signs of ACS and no history or evidence of previous shoulder surgery, shoulder trauma, previously known rotator cuff tear or labral lesion, calcific tendinosis, rheumatoid or septic arthritis, osteoarthritis > grade 1 according to the Kellgren-Lawrence classification [17], or neurologic deficit were identified in the patient population referred to our outpatient radiology institution, who all received non-enhanced and CE MRI of the shoulder on request of their referring physicians. MRIs were typically requested to rule out other causes of shoulder pain combined with limited glenohumeral ROM, to identify potential synchronous shoulder abnormalities, and in a number of cases in an attempt to confirm the clinically suspected diagnosis of ACS.

The standard of reference for clinical signs of ACS was defined as the presence of four or more of the following signs reported by experienced orthopedic surgeons and summarized in a consensus definition by Zuckerman et al. [1]: (1) increasing shoulder pain, particularly at night; limitations in active and passive shoulder range of motion (ROM) including (2) reduced anterior flexion and (3) abduction (less than 90 degrees anterior flexion and abduction, respectively), (4) reduced external (ER) and (5) internal (IR) rotation (less than 50% ER and IR of the contralateral shoulder, respectively). Clinical signs of the patients were documented by the treating physician on a standardized questionnaire. The control group consisted of 120 randomly chosen patients who were referred to our outpatient institution for non-enhanced and CE MRI in the same time period for various shoulder symptoms but had no clinical signs of ACS. Common indications for their MRI studies consisted of diagnostic workup in clinical shoulder impingement, suspected rotator cuff disease, calcific tendinosis, bursitis, and symptomatic acromioclavicular (AC) joint degeneration. In these patients, CE sequences were requested by their referring physicians, mainly to rule out inflammatory or reactive hyperemic changes. Patients with osteoarthritis > grade 1 according to the Kellgren-Lawrence classification and clear signs of synovitis were not included into the control group. All patients of the control group had MRI on the same scanners and with the same imaging protocol as patients with ACS. The control group was deliberately created with twice the number of patients of the ACS group to reduce the influence of preselection bias on the readers’ choice. In both groups, studies of patients with an age under 18 years were not included. Approval from the institutional review board was obtained. Because of the retrospective character of the study and complete anonymization of all patient-related data, no patient consent to the study was requested by the board.

### MR imaging protocol

MR imaging of all shoulder joints was performed on the same 3-T scanners (Skyra; Siemens Erlangen, Germany) using a dedicated 15-channel shoulder coil. The imaging protocol included non-CE oblique coronal T1-weighted (T1w) sequences with TR/TE of 650–700/10 ms, fat-saturated oblique coronal and axial proton density–weighted (PDw fat sat) sequences with TR/TE of 3800–4000/36 ms, and T1w fat sat oblique sagittal sequences immediately after IV contrast administration. For all sequences, a field-of-view (FOV) of 16 cm and a slice thickness of 3 mm were used.

### Image review and analysis

Image review and quantitative analysis were performed using a commercially available picture archiving and communication system (JiveX; Visus Health IT, Bochum, Germany). All MR studies were anonymized and independently reviewed by three radiologists with different experiences in musculoskeletal imaging and blinded to all clinical information. To avoid interpretation bias, ACS cases and control cases were pooled together and the order of reading the total of 180 studies was randomly assigned.

Reader 1 was a resident with 3 years of experience in radiology, reader 2 a board-certified radiologist with 8 years of experience; both readers interpreted musculoskeletal MRI studies as part of their clinical practice. Reader 3 was an expert in musculoskeletal imaging with more than 20 years of experience. In a first step, only non-enhanced sequences were evaluated and in a second step 5 weeks later, non-enhanced and CE sequences were interpreted together. In both evaluation steps, readers were asked to decide if a patient’s study shows MR imaging signs of ACS or not, respectively. Decision-making for non-enhanced sequences was based on MR imaging features which were shown to be relevant for the diagnosis of ACS according to the current literature: thickness of the joint capsule of the axillary recess (≥ 4 mm) and increased signal intensity in PDw fat sat sequences [2, 11, 15, 18–21], thickness of the capsule in the rotator interval (≥ 7 mm) [2, 12, 15, 19, 21], thickness of the coracohumeral ligament (> 3 mm), anterior capsular thickness (> 3.5 mm) [2, 14, 15, 18, 20, 22], and obliteration of the subcoracoid fat triangle [2, 14, 15, 23]. Decision-making for CE images was based on MR imaging features which were shown to be relevant for the diagnosis of ACS according to the current literature: contrast enhancement of the joint capsule in the axillary recess and in the rotator interval was used as a feature of ACS [2, 11, 12, 15, 19]. Contrast enhancement was considered positive when the joint capsule displayed higher signal as the adjacent muscle on the T1w fat-saturated sequence, as has been applied in recent studies [16, 24].

Furthermore, readers were asked to indicate their level of confidence in making or ruling out the diagnosis of ACS by using a 3-point scale (1 = uncertain, 2 = probably, 3 = certain) that was adapted from the Likert scale. This rating was a subjective assessment and independent of the presence or absence of one or more of the abovementioned MR imaging features.

### Statistical analysis

Statistical analysis was performed using R-Studio (Version 4.0.4, RStudio Inc., Boston, MA) and PRISM (GraphPad Software Inc., San Diego, CA). Differences in sensitivity and specificity between non-enhanced image sets and additional CE images as well as between readers with different experiences were assessed using McNemar’s test and paired *t* test. Confidence in making or ruling out the diagnosis of ACS was assessed using the paired Wilcoxon test for non-parametric data. The interobserver agreement among the three readers for the diagnosis of ACS was evaluated using the Fleiss kappa statistic. The degree of agreement was classified using kappa values according to the recommendation by Landis and Koch [25] as follows: 0.41–0.60, moderate agreement; 0.61–0.80, substantial agreement; 0.81–1.00, almost-perfect agreement.

A *p* value of 0.05 was set as the limit of statistical significance.

## Results

### Patients

Sixty of 180 patients had clinical signs of ACS as defined by the standard of reference for this study and different levels of imaging characteristics of adhesive capsulitis. Mean age was 58 (SD ± 12.1) years in the control group and 59 (SD ± 10.8) years in the ACS group. Ratio of women to men was 51 to 49% (61 female and 59 male patients) in the control group and 48 to 52% (29 female and 31 male patients) in the ACS group.

In the ACS group, 96.7% (58/60) presented with shoulder pain, 96.7% (58/60) with reduced external rotation, 88.3% (53/60) with reduced abduction and anterior flexion, and 96.7% (58/60) with reduced internal rotation. MRI was performed in a range of 1 to 48 weeks after onset of symptoms with a mean time interval of 13.4 weeks.

### Interobserver reliability

Interobserver agreement for the diagnosis of ACS between the readers was moderate for non-enhanced images and substantial for CE images. There was a kappa of 0.43 (95% CI, 0.34–0.51) for non-enhanced images and 0.68 (95% CI, 0.59–0.76) for CE images, respectively.

### Sensitivity and specificity of MR imaging in the detection of ACS based on non-enhanced sequences alone and with additional CE sequences

Reader 1 made the diagnosis of ACS in 37 of 60 patients (61.7%) with clinical signs of disease based on non-enhanced images alone and in 50 of 60 patients (83.3%) based on non-enhanced and additional CE images. For reader 2, the results were 33 of 60 (55.0%) for non-enhanced images and 51 of 60 (85.0%) for non-enhanced plus CE images, respectively. Reader 3 detected 45 of 60 patients with ACS (75.0%) on non-enhanced studies and 53 of 60 patients (88.3%) on both non-enhanced and CE studies together.

**Fig. 1:**
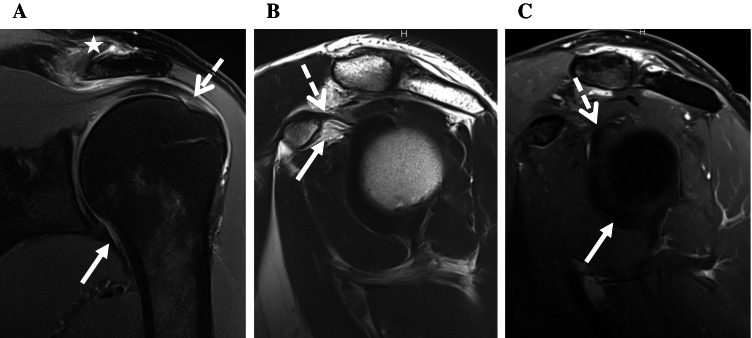
MR images from the left shoulder of a 49-year-old male with painful left shoulder but neither clinical nor MR imaging signs of ACS. **A** Fat-saturated oblique coronal PDw image shows a normal capsule of the axillary recess (arrow) without thickening or signal increase. There is tendinosis of the supraspinatus tendon (dotted arrow) and osteoarthritis of the acromioclavicular joint (star). **B** T2w oblique sagittal image demonstrates normal subcoracoid fat (arrow) as well as normal CHL (dotted arrow). **C** T1w fat-saturated oblique sagittal image after IV contrast shows no contrast enhancement of the capsule in the rotator interval (dotted arrow) and the axillary recess (arrow)

**Fig. 2:**
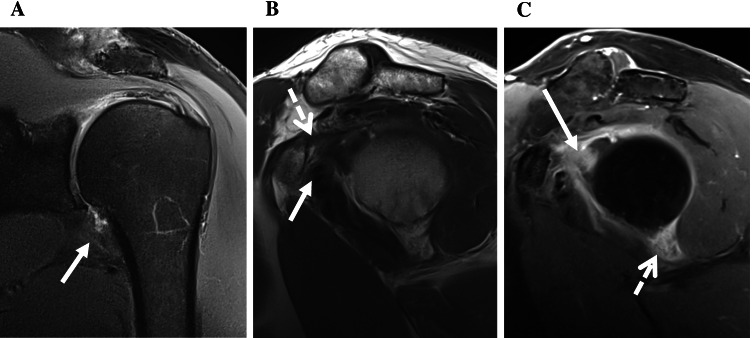
MR images from the left shoulder of a 53-year-old male with clinical signs of ACS. **A** Fat-saturated oblique coronal PDw image displays thickening and mild signal increase of the capsule of the axillary recess (arrow) indicating ACS. **B** T2w oblique sagittal image shows obliterated subcoracoid fat triangle (arrow) as well as a thickened CHL (dotted arrow) and capsule in the rotator interval. **C** T1w fat-saturated oblique sagittal image after IV contrast demonstrates capsular enhancement in the rotator interval (arrow) and axillary recess (dotted arrow)

**Fig. 3:**
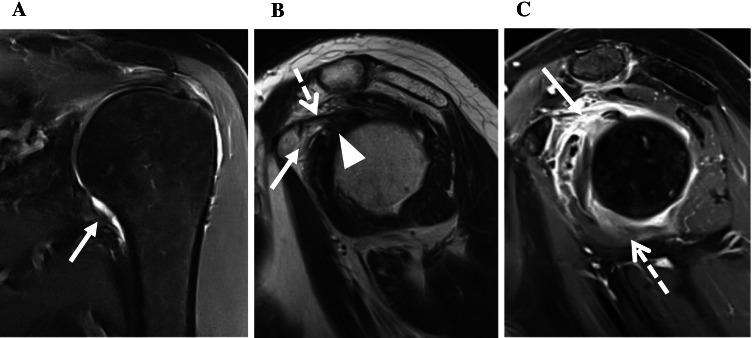
Left shoulder of a 57-year-old male with clinical signs of ACS. **A** Fat-saturated coronal PDw image showing a normal capsule of the axillary recess (arrow). **B** T2w oblique sagittal image showing minimally obliterated subcoracoid fat (arrow) as well as a not thickened CHL (dotted arrow). The capsule in the rotator interval (arrowhead) is slightly thickened up to 5 mm but does not extend the threshold value of 7 mm indicative for ACS [26]. **C** T1w fat-saturated oblique sagittal image after IV contrast with strong enhancement in the rotator interval (arrow) and moderate enhancement of the axillary recess (dotted arrow)

The sensitivity in detecting ACS improved from 61.7% with non-enhanced images to 83.3% with additional CE images for reader 1, from 55.0 to 85.0% for reader 2, and from 75.0 to 88.3% for reader 3. For each reader, sensitivity improved significantly using the McNemar test (reader 1: *p* < 0.01, reader 2: *p* < 0.001, reader 3: *p* = 0.045). Typical case examples from the control and ACS group are shown in Figs. 1, 2, and 3.

**Fig. 4:**
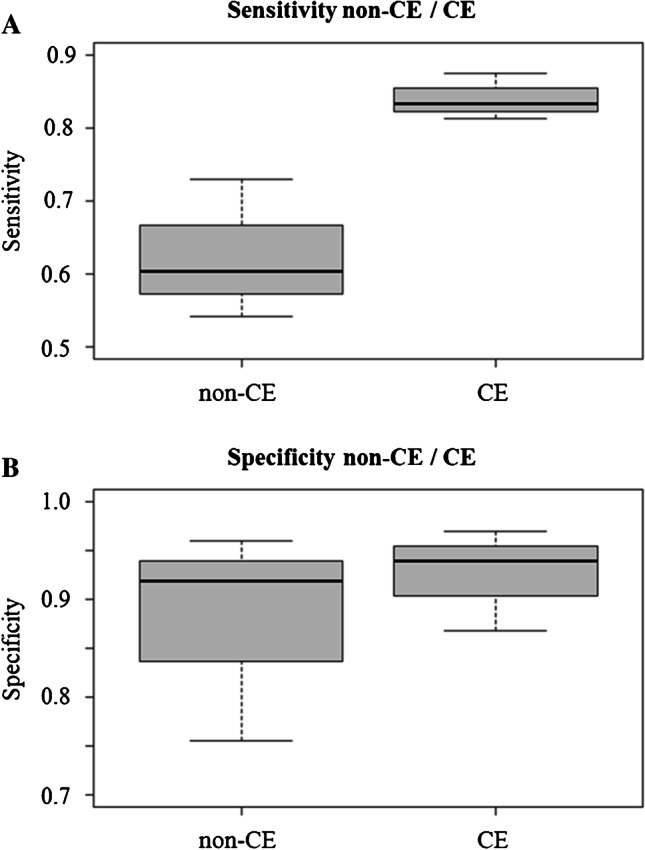
Box plots showing medians, minimum and maximum for sensitivity and specificity in the diagnosis of ACS for 3 readers based on non-enhanced MRI alone and together with additional CE sequences. **A** Graph shows sensitivity in diagnosis of ACS with non-enhanced (non-CE) images alone and together with CE images. Mean sensitivity was 63.9% for non-enhanced images and 85.5 for non-enhanced plus CE images. **B** Graph shows specificity in diagnosis of ACS with non-enhanced and additional CE images. Mean specificity for non-enhanced images alone was 86.4% and 91.9% for non-enhanced plus additional CE images

The mean sensitivity determined for all three readers together increased significantly from 63.9% using non-enhanced images to 85.5% with additional CE sequences using the paired *t* test (*p* = 0.046; Fig. 4).

Reader 1 identified 93 of 120 patients without ACS correctly using non-enhanced images and 114 of 120 using non-enhanced plus CE images. Reader 2 identified 106 of 120 patients without ACS correctly with non-enhanced and 102 of 120 patients with additional CE sequences. The respective identification rates of reader 3 for patients without ACS were 112 of 120 (non-enhanced images) and 115 of 120 (non-enhanced plus CE images).

The specificity for detection of ACS increased from 77.5% with non-enhanced images to 95.0% with additional use of CE images for reader 1, decreased slightly from 88.3 to 85.0% for reader 2, and improved from 93.3 to 95.8% for reader 3. Only for reader 1 the specificity improved significantly using the McNemar test (*p* < 0.001). For readers 2 and 3, no statistically significant difference in specificity was found with additional use of CE images. The mean specificity determined for all three readers together showed no significant difference for the use of non-enhanced images alone (86.4%) and for the use of both non-enhanced images and CE images together (91.9%; *p* > 0.05) (Fig. 4).

### Influence of reader´s experience on the MR imaging diagnosis of ACS

As described above and displayed in Table 1, reader 3 with more than 20 years of experience in musculoskeletal imaging achieved higher values of sensitivity and specificity for both non-enhanced images alone and non-enhanced plus CE images than less experienced readers 1 and 2. A statistically significant difference in sensitivity was found between readers 2 and 3 with non-enhanced images (*p* < 0.01). For specificity, a statistically significant difference was found between readers 1 and 2 for non-enhanced (*p* < 0.05) and between readers 1 and 3 as well as readers 2 and 3 for CE images (reader 1/2: *p* < 0.001; reader 2/3: *p* < 0.01).

**Table 1:** Readers’ sensitivity and specificity

		Sensitivity (in %)	Specificity (in %)
Reader 1	Non-enhanced	61.7	77.5
	Non-enhanced + CE	83.3	95.0
Reader 2	Non-enhanced	55.0	88.3
	Non-enhanced + CE	85.0	85.0
Reader 3	Non-enhanced	75.0	93.3
	Non-enhanced + CE	88.3	95.8

Values indicate sensitivity and specificity (columns) of all readers for non-enhanced and CE images (rows)

### Effects of additional CE MR images on reader’s confidence in the diagnosis of ACS

**Fig. 5:**
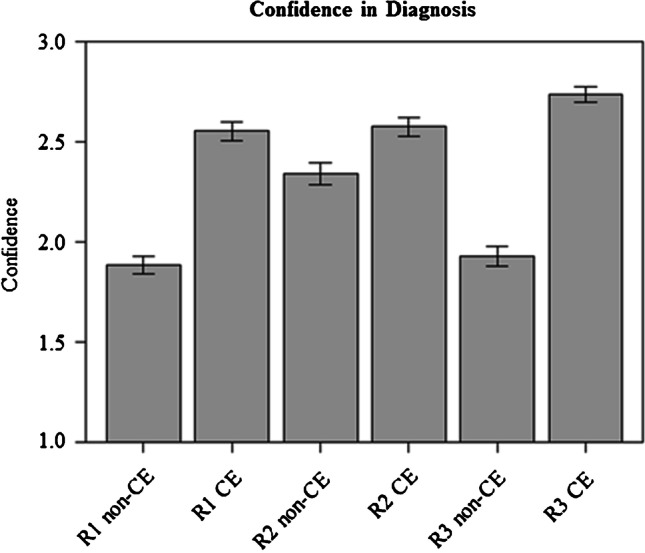
Subjective confidence in diagnosis of ACS. Graphs show plots for readers’ (reader 1: R1; reader 2: R2; reader 3: R3) subjective confidence in MR-based diagnosis of ACS based on our 3-point scale. For each reader, confidence increased significantly between non-enhanced (non-CE) and non-enhanced plus CE images (*p* < .001 for readers 1 and 3 and *p* < .01 for reader 2)

The effects of additional use of CE images on the reader’s subjective confidence in diagnosing or ruling out ACS are summarized in Table 2 and graphically displayed in Fig. 5. Reader 1 subjectively considered his diagnosis (ACS vs. no ACS) as “certain” in 23 of 180 patients using non-enhanced images and in 109 of 180 patients using additional CE images. Reader 2 considered the diagnosis as “certain” in 91 of 180 patients using non-enhanced images and in 118 of 180 patients using additional CE images. The respective results for reader 3 were 32 of 180 patients and 139 of 180 patients.

**Table 2:** Confidence in diagnosis of ACS

	Uncertain	Probably	Certain	Total
R1 non-enhanced	44	113	23	180
R1 non-enhanced + CE	10	61	109	180
R2 non-enhanced	30	59	91	180
R2 non-enhanced + CE	16	45	119	180
R3 non-enhanced	45	103	32	180
R3 non-enhanced + CE	6	35	139	180

Values indicate how often each level of confidence (columns) was rated by every reader for non-enhanced and non-enhanced plus CE images (rows)

For reader 1, the mean value for confidence was 1.9 (grading of confidence: 1 = uncertain; 2 = probably; 3 = certain) with non-enhanced images and 2.6 for non-enhanced and CE images; for reader 2, mean value for confidence was 2.3 with non-enhanced images and 2.6 for CE images; and for reader 3, the respective values were 1.9 with non-enhanced images and 2.7 for CE images. For all readers, differences of their confidence values between non-enhanced and additional CE sequences were statistically significant (reader 1: *p* < 0.001; reader 2: *p* < 0.01; reader 3: *p* < 0.001).

## Discussion

ACS has been defined primarily as a clinical diagnosis based on history and physical examination [22], which is characterized by multidirectional restriction of both active and passive shoulder motion [1]. So far, the major role of imaging in the diagnostic workup of ACS is to exclude other pathologies which could cause similar symptoms, like calcific tendinosis or rotator cuff disease, as well as to help in confirming the diagnosis of ACS in unclear cases [1, 2]. Especially in clinically equivocal cases, high sensitivity and specificity of MR imaging may substantially contribute to establish the correct diagnosis in order to guide further treatment decision.

In this retrospective study, 180 shoulder MRIs were evaluated with regard to the presence or absence of ACS by three readers with different grades of experience in musculoskeletal imaging. The cohort included 60 patients with clearly defined clinical signs of ACS and 120 patients without these signs but a variety of other shoulder abnormalities such as impingement syndrome, rotator cuff disease, calcific tendinosis, and acromioclavicular osteoarthritis. To our knowledge, our study is the largest study on MR imaging–based diagnosis of ACS and the first study that investigated CE MRI versus non-enhanced MRI of the shoulder for the confidence level of the diagnosis. Furthermore, we used an unambiguous definition of the standard of reference for this disease consisting of the presence of at least four out of five typical clinical symptoms.

In our study, the diagnosis was made by the readers in consideration of all imaging signs displayed in a patient’s MRI data set and on purpose not on the presence or absence of one or more specific signs of ACS as none of these individual signs was shown to be perfectly sensitive or specific [7, 11, 14, 18, 20–22, 24]. Moreover, for some of these imaging signs various cutoff values were published in the literature indicating potential limitations of their discriminatory power. For example, for thickening of the CHL as a sign of ACS, Mengiardi et al. found the highest accuracy using a cutoff of 4 mm [26] while Lee et al. found a cutoff of 3 mm more accurate [18]. There has been also debate about the usefulness of joint capsule thickness measurements in the axillary recess for the diagnosis of ACS. While Emig et al. [21] reported that capsular thickening of more than 4 mm in the axillary recess was a useful criterion for the diagnosis of ACS, Mengiardi et al. [26] did not find significant thickening of the axillary recess in ACS.

Thus, the primary objective of previous studies was to establish imaging signs of ACS and specific cutoffs; however, the clinically relevant radiologic differentiation whether a subject suffers from ACS or not was not the main focus.

Independent from reader’s experience, in our study the sensitivity of MR imaging in the diagnosis of ACS increased significantly for all readers with additional use of CE images as compared to non-enhanced images alone. Similarly, subjective confidence in their diagnosis increased significantly for all readers when additional CE images could be used. Experience in musculoskeletal imaging had a positive effect on the correct diagnosis of ACS using non-enhanced images alone or together with additional CE images. The positive effect, however, was statistically significant only for some of the constellations investigated in our study.

The reader’s decision was based on MR imaging signs attributed to ACS in the current literature, such as thickening of the joint capsule in the rotator interval and in the axillary recess, signal alteration of the capsule in the axillary recess in non-enhanced PDw fat-saturated images, thickening of the coracohumeral ligament, obliteration of the subcoracoid fat triangle, and contrast enhancement of the rotator interval and the axillary recess, respectively [2]. Interestingly, our results are not completely in line with previous findings: Ahn et al. [11] found none or only marginal improvement of accuracy for CE-based parameters like enhancement of the axillary capsule (sensitivity 96–98%, specificity 64–66%) as compared to parameters based on non-enhanced images like axillary capsular thickening and hyperintensity (sensitivity 84–94%, specificity 64–74%). In contrast, we found in our study a statistically significant improvement in mean sensitivity from 63.9% with non-enhanced images alone to 85.5% when non-enhanced images were interpreted together with CE images. Mean specificity increased in our study from 86.4 to 91.9%; however, this improvement did not show statistical significance. This might be related to the fact that in our setting the diagnosis was established by the readers in consideration of all imaging signs displayed in a patient’s study instead of the presence or absence of only one or few individual signs of ACS. Moreover, in the study of Ahn et al. [11] cutoffs were based on the differentiation of no vs. mild enhancement which might explain their reported high sensitivity and poor specificity. On the other hand, the sensitivity found for our readers based on non-enhanced images corresponds approximately with that in other studies, e.g., of Chi et al. [14] who found sensitivities of 23.3 to 76.7% for various parameters in non-enhanced images. Song et al. [27] found a higher sensitivity in the detection of ACS for contrast enhancement of the axillary recess (91%) compared to findings in T2w sequences (69%). However, these authors used indirect MR arthrography (MRA) to obtain CE images, which required active joint movement after IV contrast application and a delay of 15 min between contrast application and the post-contrast scan. We did not consider indirect MRA as the preferable technique of CE imaging because delayed post-contrast scanning may be accompanied by diffusion of contrast medium into joint fluid, thus affecting the delineation of capsular tissue from joint fluid [28].

Our results show that sensitivity and specificity in the diagnosis of ACS increased with reader’s experience and contrast administration. Additional use of CE images was particularly beneficial for the improvement of diagnostic accuracy in readers with less experience in musculoskeletal imaging. Furthermore, interobserver reliability between all readers was higher with additional use of CE images compared to non-enhanced images alone. This may have some relevance for the interpretation of MR studies in daily routine settings, when studies cannot always be interpreted by a musculoskeletal expert reader.

ACS is generally a self-limiting disease, in which, particularly in initial stages, physiotherapy and pharmacological therapies consisting of non-steroidal anti-inflammatory drugs (NSAIDs) and systemic or intra-articular corticosteroids are recommended [22]. Therefore, ACS should not be confounded with other shoulder diseases that may require surgery. Considering the significantly improved sensitivity and the moderately improved specificity of MRI in the workup of patients with suspected ACS by using additional CE sequences, these seem to be justified at least in patients whose clinical presentation is unclear with respect to ACS.

The finding of an insignificantly decreased specificity of reader 2 with additional use of CE sequences contrary to readers 1 and 3 is difficult to interpret and might be considered an extraordinary random statistical result.

ACS usually presents with a four-staged progression as initially described by Neviaser [2, 3]. Especially in stages 1 and 2, patients suffer from pain which usually diminishes in stages 3 and 4, whereas restricted range of motion has its maximum in stages 2 and 3 and in some cases persists beyond stage 3. The early pain-associated stages usually show an erythematous and thickened synovium in arthroscopy [2, 26]. Several studies tried to correlate these clinical stages which MR findings. Ahn et al. found an association of gadolinium enhancement of the joint capsule in the axillary recess with shoulder pain [11] and Sasanuma et al. found a significantly increased pain score in patients with enhanced deposition of contrast medium in the axillary pouch compared to patients with reduced deposition of contrast medium [29]. This is accompanied by inflammatory processes, as in stage 1 an infiltration of inflammatory cells in the synovium and in stage 2 synovial proliferation was detected histologically [22]. Moreover, immunohistochemically increased expression of several fibroblast activation markers was found [10]. As 96.7% of the ACS population in our study suffered from shoulder pain, most patients would probably be assigned to stages 1–3. MRI studies were obtained 1–48 weeks after onset of symptoms with a mean of 13.4 weeks which can predominantly also be assigned to stages 1 and 2. Hence, we assume that especially in stages 1–3, where pain and inflammation of the synovium and joint capsule are dominant, accuracy may improve more with CE sequences than in stage 4.

Our study has several potential limitations. One limitation lies in the retrospective character of our study. Furthermore, as many patients of our study population received conservative treatment, which is the standard therapy for ACS, we were not able to obtain arthroscopic or histological correlation of clinical and MR morphological findings in most cases. Another limitation of our study is that due to the clearly designed clinical standard of reference including the presence of at least four out of five clinical signs of ACS, we have probably included almost exclusively cases with clinically clear diagnoses of ACS but not clinically difficult cases. This potential limitation, however, reveals the dilemma underlying all studies on ACS, as no true “golden standard” other than histology has been defined for this usually self-limiting and conservatively treated disease, for which tissue sampling is not commonly recommended. It should also be mentioned that administration of contrast agent may have implications in relation to extra costs, longer scanning time, and patients’ convenience. In our department, scanning time is increased up to 5 min when CE sequences are added.

We conclude that additional contrast-enhanced sequences can significantly increase the sensitivity of MRI as well as radiologist’s confidence in establishing the diagnosis of ACS. In addition, we found that reader’s experience improves the accuracy of MRI in the detection of ACS. For the specificity of MRI in diagnosing ACS, additional CE images may not provide a clinically relevant benefit. Thus, we recommend additional CE sequences in doubtful cases to improve the MR imaging diagnosis of ACS.
